# Portable impedance-sensing device for microorganism characterization in the field

**DOI:** 10.1038/s41598-023-37506-1

**Published:** 2023-06-29

**Authors:** Karim Bouzid, Jesse Greener, Sandro Carrara, Benoit Gosselin

**Affiliations:** 1 Department of Electrical and Computer Engineering, Laval University, Quebec-City, G1V 0A6 Canada; 2 Department of Chemistry, Laval University, Quebec-City, G1V 0A6 Canada; 3 Institute of Electrical and Micro Engineering, École Polytechnique Fédérale de Lausanne (EPFL), 1015 Lausanne, Switzerland

**Keywords:** Electrical and electronic engineering, Flow cytometry, Classification and taxonomy

## Abstract

A variety of biosensors have been proposed to quickly detect and measure the properties of individual microorganisms among heterogeneous populations, but challenges related to cost, portability, stability, sensitivity, and power consumption limit their applicability. This study proposes a portable microfluidic device based on impedance flow-cytometry and electrical impedance spectroscopy that can detect and quantify the size of microparticles larger than 45 µm, such as algae and microplastics. The system is low cost ($300), portable (5 cm $$\times$$ 5 cm), low-power (1.2 W), and easily fabricated utilizing a 3D-printer and industrial printed circuit board technology. The main novelty we demonstrate is the use of square wave excitation signal for impedance measurements with quadrature phase-sensitive detectors. A linked algorithm removes the errors associated to higher order harmonics. After validating the performance of the device for complex impedance models, we used it to detect and differentiate between polyethylene microbeads of sizes between 63 and 83 µm, and buccal cells between 45 and 70 µm. A precision of 3% is reported for the measured impedance and a minimum size of 45 µm is reported for the particle characterization.

## Introduction

Microorganisms are ubiquitous in nature, being found in environments such as lakes, soils, plants, and within animals. Some are involved in well-known bioprocesses such as fermentation in the food and drink industry, and more recently antibiotics and biofuels. New applications are currently researched in the field of biotechnology, with goals to degrade synthetic plastics^1–3^, regularize emotions and stress responses using gut microorganisms^4,5^, monitor climate change and natural habitats^6–8^, remediate nuclear wastes^9^, detect buried landmines^10^, or judge of the water quality of popular beaches based on the presence of large phytoplankton that produce neurotoxins such as *Karenia brevis*, *Alexandrium fundyense*, *Dino-physis acuminata*, and *Pseudo-nitzschia*^11^. However, despite their utmost importance and numerous applications to human and ecological activities, the vast majority of microorganisms are currently not catalogued, their existence having been only extrapolated from the results of recent phylogenetic studies and genomics^12,13^. Sophisticated sensors and equipment and a thorough understanding of physics, genomics, optics, taxonomy, and biology are necessary to test, characterize, and classify microorganisms, and a wide array of properties can be tested using different bioreceptors^14–16^. Studying microorganisms is thus time-consuming and costly, added that microorganisms are too small to be studied with the bare eyes and mutate at a considerably faster rate than animals and plants, making it difficult to characterize them across time^12,17,18^. Moreover, replicating their heterogeneity, motility and unique behavior in laboratory settings is found to be challenging, especially considering their extreme sensitivity to their environment, where a minute variation in humidity, light intensity, pH, or temperature is enough to stunt the growth of entire populations^19^. The more resilient microorganisms are the ones most studied in the literature, the best example being the well-known Escherichia coli.

Following these challenges, the objective of this study is to conceive a portable intelligent biosensor to characterize multiple properties of large microorganisms and microparticles autonomously and directly in their own natural habitat^14^. The device should be autonomous, requiring little to no supervision. Automated operations should include the retrieval of the important parameters of hundreds to thousands of microparticles per seconds. This will lead to a high-throughput technique to characterize and differentiate between microorganisms and microparticles polluting the ecosystems. A broad range of approaches currently exist for the characterization and study of microorganisms, including imaging and hyperspectral-based solutions^20–22^, mass spectroscopy^23^, specialized biochemical sensors^15,24^, and flow-cytometry^25^. Impedance-based measurements, especially when combined with electrical impedance spectroscopy (EIS)^26,27^ and impedance flow cytometry (IFC)^28,29^ seems especially promising.

The common way to monitor impedance is to use commercial benchtop instruments. However, those are generally too expensive and bulky for portable applications. Certain commercial LCR meters offer high precision impedance measurement with errors under 0.5%, but those units are costly, high power consuming, heavy, and bulky, which makes them unpractical for high-volume portable applications. Market-available impedance analyzers can be found in portable formats, but their prices are prohibitive for large scale deployment. As an alternative to these instruments, low-power low-cost integrated chips exist with impedance analyzer capabilities^30–33^. These chips can be used as all-in-one-package solutions for low-cost impedance analysis, but are not as versatile as benchtop instruments and their excitation frequency often proves insufficient for microorganism characterization. Other portable impedance analyzers reported in Table 1 exist in the literature, based on techniques such as digital-signal-processing (DSP) sine-fitting^34^, direct digital synthesizer (DDS) EIS^35^, mixed analog/digital lock-in amplifier (LIA)^36^, indirect Kramers–Kronig transformation^37^, but none of these solutions is a perfect match for high-throughput microparticle characterization.

To fill this gap, we present here a low-cost portable impedance biosensor which improves the authors previous sensor design^38,39^ and concepts from printed circuit board integrated directly in a microfluidic device^40^. The presented device can autonomously monitor the impedance of large microorganisms at a high throughput directly in their own natural habitats without using any harmful chemicals, and determines their characteristics based on their impedance profile using EIS and IFC. The main novelty of the device is found in its square wave excitation signal and quadrature phase-sensitive detectors (PSDs). It is used with an algorithm to compensate for the high-level harmonics introduced with the square wave signal.

**Table 1:** Impedance analyzers found in the literature.

Source	Design	Frequency range	Impedance range	Precision (%)	Portable?	Cost
LCR-6002	LCR meter	< 300 kHz	< 300 M$$\Omega$$	0.5	No	$1200
µStat-i-400s	Impedance analyzer	1 mHz–1 MHz	5 $$\Omega$$–350 M$$\Omega$$	0.5	Yes	$10 k
PalmSense4	Impedance analyzer	100 uHz–1 MHz	1 $$\Omega$$–3 G$$\Omega$$	1	Yes	> $10 k
Al-Ali^31^	AD5933	5 Hz–100 kHz	10 $$\Omega$$–100 k$$\Omega$$	10	Yes	$150
Sylvain^32^	AD5933	750 Hz–10 kHz	80 $$\Omega$$–12 k$$\Omega$$	2.35	Yes	$500
Chowdhury^35^	DDS-based	50 Hz–100 kHz	100 $$\Omega$$–20 k$$\Omega$$	10	Yes	$1000
Radil^34^	DSP sine-fitting	< 12.5 kHz	1 k$$\Omega$$–10 k$$\Omega$$	0.85	Yes	?
Allegri^36^	Hybrid LIA	10 kHz–10 MHz	1 $$\Omega$$–1 k$$\Omega$$	0.8	Yes	?
Al-Ali^37^	Kramers–Kronig	1 Hz–10 MHz	100 $$\Omega$$–280 k$$\Omega$$	8	Yes	$95
This work	Square waves PSDs	70 kHz–12 MHz	200–$$\Omega$$ 120 k$$\Omega$$	<3	Yes	$300

## Principles of impedance-flow cytometry

The characterization of microorganisms can be performed using their impedance spectrum, which is a function of the resistivity, dielectric constant, and geometry of the substance under test (SUT). The resistivity and dielectric constant depends on the mobility and quantity of charge carriers in the material^41^. The impedance is defined from the complex Ohm’s law based on the ratio of a voltage signal to a current signal^37^, as shown in Eq. 1, where *Z* is the impedance, *V* is the applied (or measured) voltage, *I* is the measured (or applied) current, $\phi$ is the phase difference between the voltage and current, $\omega$ is the angular excitation frequency, and *j* is the imaginary unit value. EIS measurement consists in injecting an AC sinusoidal waveform of a known voltage or current to the SUT and measuring its respective output current or voltage response, for a certain number of frequency.

1 $$\begin{aligned} Z(j\omega ) = \frac{V_{in}(j\omega )}{I_{out}(j\omega )} = \frac{V_{out}(j\omega )}{I_{in}(j\omega )} = \frac{|V |}{|I |} \times e^{-j\phi } = |Z |e^{-j\phi } = \Re (Z) +j \times \Im (Z) \end{aligned}$$

The permittivity of the medium, membrane, and cytoplasm are fundamental properties of cells, making them suitable for microorganism characterization. The simplest model for a biological cell with a lipid layer plasma membrane is the single-shell model^28,42–46^ modelled by a homogeneous phase cytoplasm and an insulating thin shell. Bacteria, yeast, and plant cells are examples of microorganisms that possess a cell wall outside of the plasma membrane. This addition modifies the single shell model to a double shell model. An in-depth cells analysis and modeling strategy is available in (Asami, 2002)^42^.

**Figure 1:**
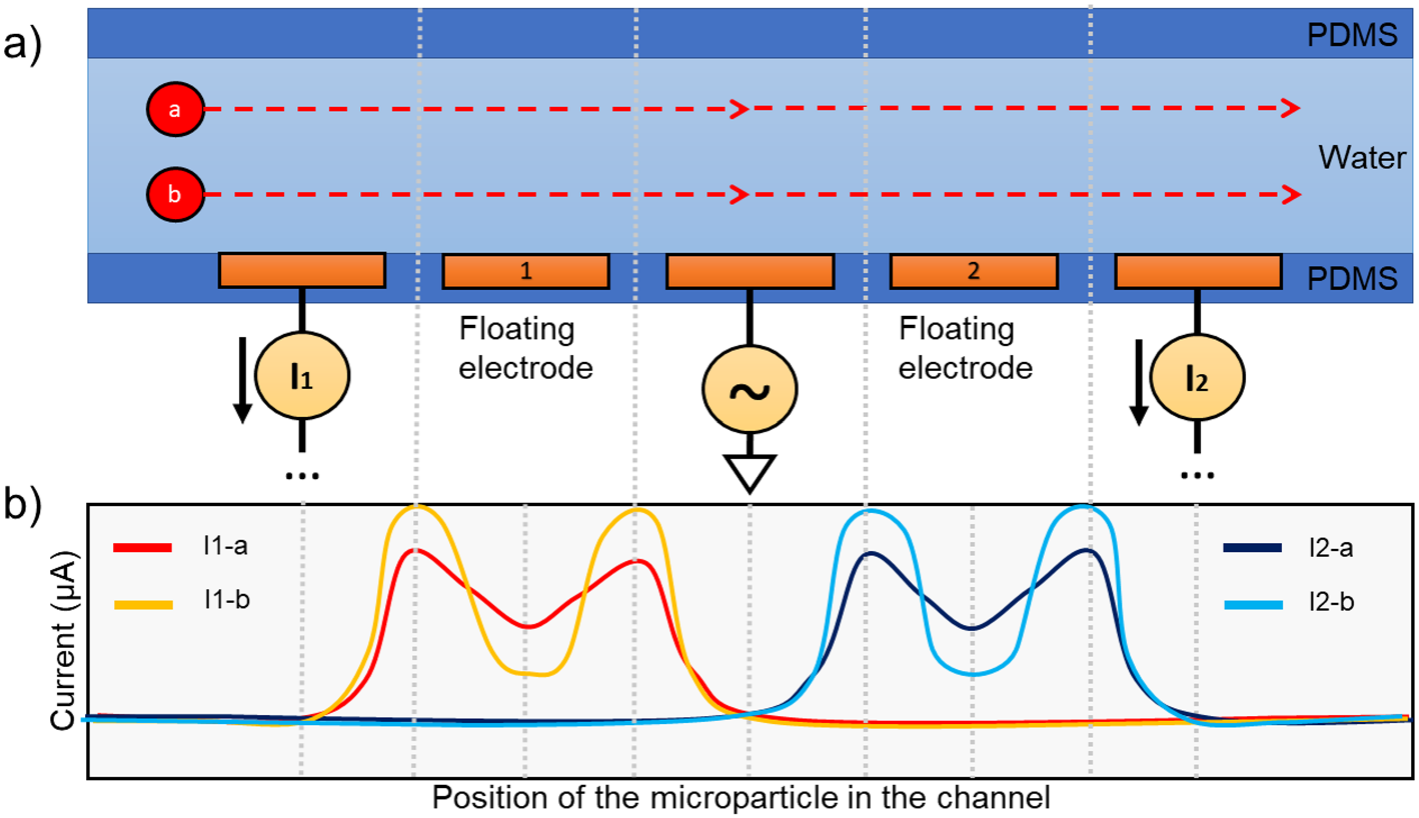
Two microparticles named **a** and **b** submerged in a liquid flow from left to right at two different height in a microchannel. (**a**) Five-electrode configuration proposed by De Ninno^51^. (**b**) Observed current responses at the first and second pair of electrode based on the position of the two microparticles **a** and **b**, with which the impedance and permittivity can be calculated.

IFC^41,44,46,47^ is the technique offering the best results in the literature for whole cell characterization so far. It is a label-free non-invasive impedimetric measurement technique based on the Coulter counter^48^ to measure the volume displacement of particles flowing in a fluid. The particles are detected using IFC by monitoring the impedance changes observed each time a particle passes over electrodes in a narrow channel. This is the case since the particles and fluid have different impedances. Figure 1 describes the principles of IFC. Electrodes are positioned on the walls of a microchannel and the impedance of a liquid flowing within is measured for several excitation frequency. A pulsed waveform resembling the one shown at the bottom of Fig. 1 is thus retrieved for each frequency when a cell flows in the channel. The shape of this pulse depends on the position and relative impedance of the microparticle and fluid in the channel, while its measured module and phase depends on the volume (or size) of the particle compared to the dimensions of the channel. When used as a spectroscopy with multiple excitation frequencies, it is possible to adequately characterize the dielectric properties of the cells flowing inside a microchannel, which can be used in association with their impedance models to retrieve important characteristics of microorganisms^28,29,45,49^. The impedance is generally converted to a complex permittivity since the geometrical parameters of the microchannel and electrodes are known, and used for the characterization. This is a simple and effective technique to count and characterize particles in a fluid, providing information in real-time for feedback control, or data for later analysis or post-processing^50^.

The high volume factor between the cell and detection area when using coplanar electrodes in IFC creates a fringing effect between the electrodes, which is difficult to modelize. A simple empirical equation is given in^51^ and^52^ as an alternative to estimate the size of microparticles when using IFC. The particle diameter *D* can be estimated using a fit to the cubic root of the combined measured impedance magnitude difference $|\Delta Z_{1} |$ and $|\Delta Z_{2} |$ observed when a particle passes the first and second electrode pairs in the channel, respectively. This is shown in Eq. 2, where *G* is a constant that accounts for all the parameters linked to the electronics and fluidics, such as the electrode configurations, magnitude and frequency of the excitation signal, filter bandwidth, channel depth and width, electrodes width, EDL capacitance $C_{edl}$, buffer conductivity, and electronics gains. The constant *G* can be determined empirically by testing the IFC system with circular beads of know diameters, then adjusting *G* until the estimated diameters match the effective beads diameters used during the test.

2 $$\begin{aligned} D = G (|\Delta Z_{1} |+ |\Delta Z_{2} |){^{1/3}} \end{aligned}$$

From the Randles^53^ model and from the impedance model of single-shelled cells^42,43^, it is possible to deduce the optimal excitation frequency range for microorganism characterization, which is found to be between 100 kHz and 10 MHz. For frequencies lower than 100 kHz, the sensibility of the sensor to microparticles is reduced considering that the electrical double-layer (EDL) and ionic diffusion from the Warburg element dominate the measured impedance^38,44,46^. Above 10 MHz, the PCB dielectric begins to shunt the channel impedance, and the parasitics of the measurement electronics significantly reduces the precision of the results. Above 1 GHz, the dionic reorientation of water molecules also affect the measured impedance. Calibration algorithms can be used to compensate for the errors obtained when using excitation frequencies outside of this range.

## Material and methods

The design and fabrication of the impedance-sensing system and microfluidic system will be described in this section based on the principles of IFC.

### Impedance-sensing system

**Figure 2:**
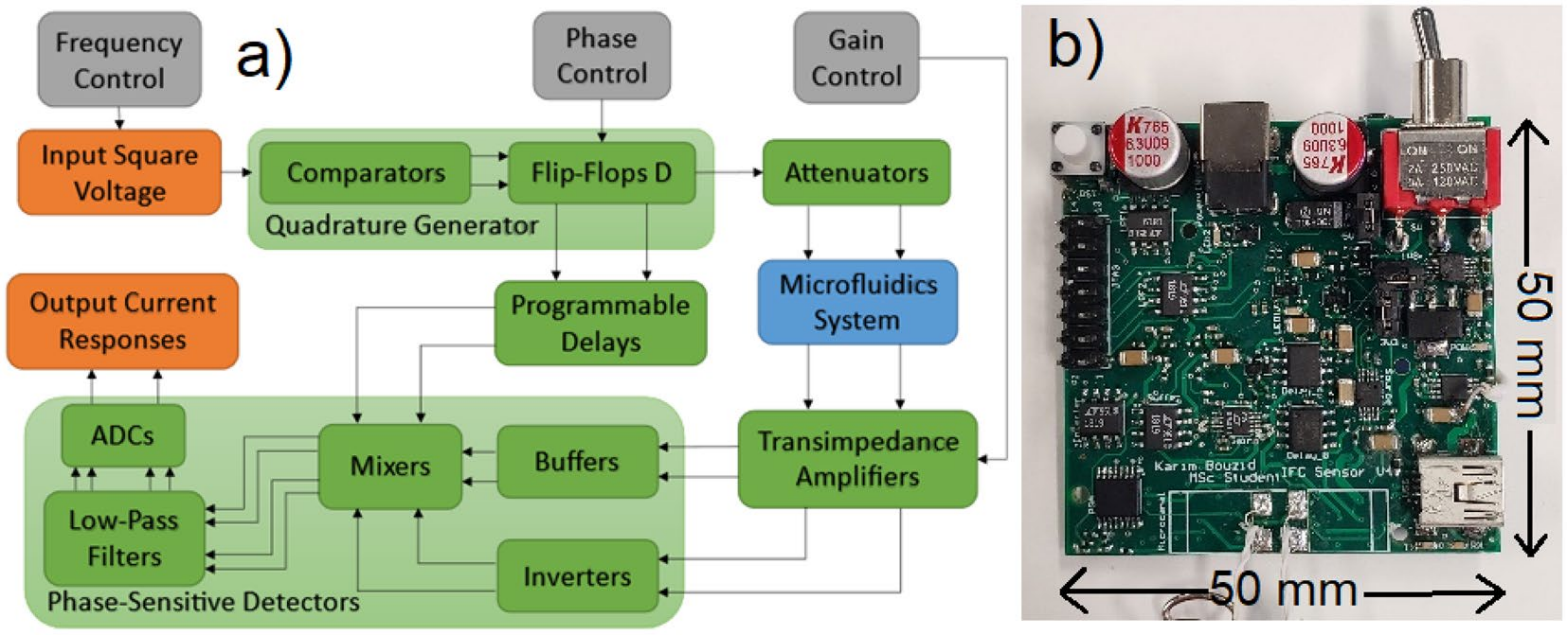
(**a**) Block-diagram and (**b**) PCB of the impedance-sensing device.

The bloc-diagram of the impedance-sensing device is shown in Fig. 2. It is based on a LIA topology, and extracts the amplitude and phase of a high frequency input signal. It features two channels used to perform a differential analysis. A square waveform with frequency ranging from 200 kHz to 200 MHz is created by the clock-system (Si5351, Silicon Lab). This signal is sent to a quadrature generator to create two 90-degree phase-shifted square waveforms at half the clock signal frequency. The in-phase waveform is attenuated to 100 mVpp to keep a safe linear current response that will not harm the cells during the experiment, and then sent to the two differential electrode pairs of the microfluidics system. Two current responses are obtained, which are then amplified and converted to voltage signals by transimpedance amplifiers (TIAs). The outputs of the TIAs are mixed by two phase-sensitive detectors (PSDs). This yields four output signals representing the real and imaginary current responses of two electrode pairs, which can be sampled by the ADCs. The impedance magnitude and phase differences can be retrieved from the real and imaginary impedance components, and are processed by a square-to-sine spectroscopy algorithm to accurately retrieve the cells properties at a high throughput. The differential design is important to decrease the effects of the common noise and increase the sensitivity to the flowing particles^46^.

#### Square input signal

IFC and EIS systems are generally performed using sinewaves. The main advantage of square-waves over sinewaves is that they replace the complex hardware associated with conventional LIAs with much simpler clock-based circuitry^54^. For instance, the digital-to-analog converter (DAC), wave generator, and linear mixer can all be replaced by a simple clock-system and inexpensive controlled switches. This leads to a decrease of the system’s power-consumption and cost, which are of a prime importance in portable applications.

The square wave input voltage $V_{in}$ outputted by the clock-system and sent to the electrodes is defined in the time domain by Eq. (3). Square signals are multi-frequency signals. Looking at their Fourier transform, we see that a square wave is composed of a fundamental frequency followed by odd-frequency harmonics of decreasing amplitudes. An ideal symmetrical square wave of amplitude $2 V_o$ with peaks at $-V_o$ and $+V_o$ follows the geometrical sum of Eq. (3).

3 $$\begin{aligned} V_{in}(t) = \frac{8}{\pi }V_o \displaystyle \sum _{n=1,3,5,7...} ^{\infty } \frac{sin(n \omega t)}{n} \end{aligned}$$

An important point to note from Eq. (3) is that the amplitude of the fundamental differs from the one of a sinewave of amplitude $2 V_o$ by a factor of $\frac{4}{\pi }$. Such a difference biases the measurements and affects the precision but can be mostly corrected (up to a couple of percents) by following an algorithm proposed by Subhan^54^. Another consideration is the introduction of harmonics in the circuit, which raises the noise floor of the system.

#### Transimpedance amplifiers

**Figure 3:**
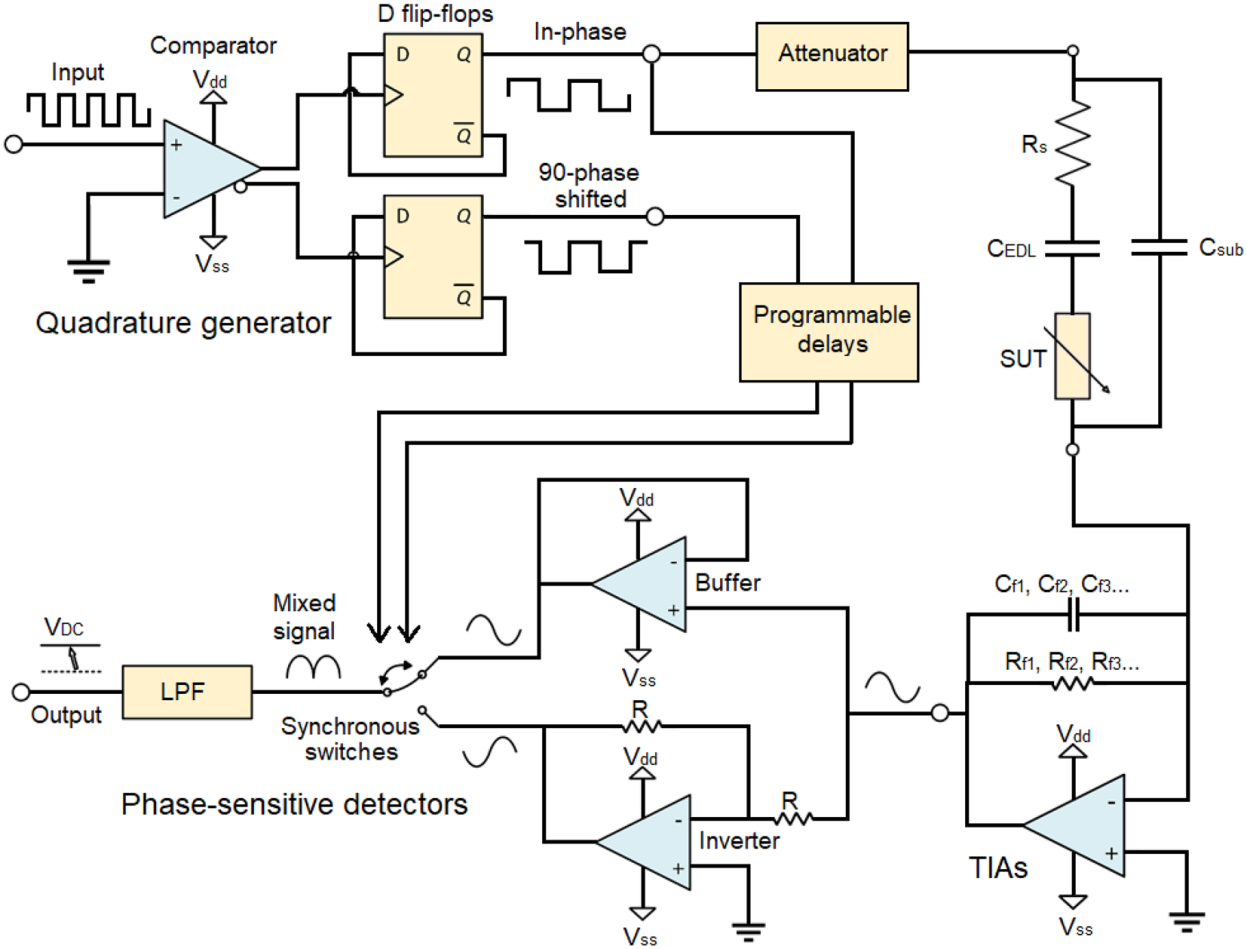
The electronics circuits of the impedance-sensing device.

Current responses are obtained from the two electrode pairs that follow Ohm’s law for complex impedance. Current responses being difficult to interact with, two transimpedance amplifiers (TIAs) are used to convert them to voltage signals. TIAs are current to voltage converters generally implemented using one operational amplifier, as shown in Fig. 3. The practical implementation also uses a capacitor for stability in parallel with the resistor in the feedback loop. In the simplest case for a square input signal, Eq. (4) represents the voltage output of the TIAs in time $V_{TIA}(t)$, where $|Z |$ and $\theta$ are respectively the impedance magnitude and phase of the SUT for any harmonic angular frequency $n \omega _0$.

4 $$\begin{aligned} V_{TIA}(t) = -\frac{4}{\pi } V_0 R_f \displaystyle \sum _{n=1,3,5,7\ldots } ^{\infty } \frac{\sin (n \omega _0 t)}{n|Z |(n \omega _0 t + \theta )} \end{aligned}$$

To accomodate for a wide range of input impedances, a programmable gain array (PGA) with a feedback resistor $R_f$ and capacitor $C_f$ is added to the TIAs to control the gain at will. The PGA is achieved using a multiplexer toggled by a microcontroler that can switch between different gain resistors in the feedback loop of the TIAs^30^. The feedback capacitor is needed by the TIAs to prevent high frequency ringing. This can cause a limitation for high frequency measurements since an attenuation is expected at frequencies higher than a couple of megahertz because of the time-constant of the RC network formed by $R_f$ and $C_f$. However, the prototype can still be used at higher frequencies with an adequate calibration, although with a reduced accuracy associated with the lessened measured signal amplitude. The trade-offs associated with TIAs are described in Orozco^55^

#### Phase-sensitive detectors

The TIAs outputs cannot be sampled directly using an ADC since the frequency of the signals of interest is too high (the relevant harmonics can go as high as 110 MHz considering the five first harmonics of a 10 MHz square signal). A solution consists of using a mixing and filtering stage implemented from a phase-sensitive detector (PSDs). PSDs act as narrowband filter similarly to LIAs to precisely retrieve the amplitude and phase of a signal buried in noise^41^. PSDs use square signals and an inverter to switch between the original and inverted version of the signal of interest at the frequency of the square reference signal. This switching yields DC components proportional to the real and imaginary current of the SUT’s impedance. The behavior and implementation of the PSD is shown in Fig. 3. The DC values of the real and imaginary components of the current responses at the output of the PSDs are described by Eqs. (5) and (6).

5 $$\begin{aligned} \Re (V_{PSD-\omega _0})= & {} \frac{1}{2} \frac{6}{\pi ^2} V_0 R_f \displaystyle \sum _{n=1,3,5,7\ldots } ^{\infty } \frac{\cos (\theta (n \omega _0))}{n^2|Z |(n \omega _0)} \end{aligned}$$

6 $$\begin{aligned} \Im (V_{PSD-\omega _0})= & {} \frac{1}{2} \frac{6}{\pi ^2} V_0 R_f \displaystyle \sum _{n=1,3,5,7...} ^{\infty } \frac{\sin (\theta (n \omega _0))}{n^2|Z |(n \omega _0)} \end{aligned}$$

#### Quadrature generator

Operating the PSDs mixers requires two square signals in quadrature. Those signals can be precisely obtained from a quadrature generator circuit using a comparator and two D-Flip-Flops, as shown in Fig. 3. This technique is ultrawideband and relatively simple to implement but can be used only for low-power binary signals since the current is sunk directly from the low-power flip-flops. Programmable delays are added in the path of the reference signals to compensate for the delays of the TIAs circuits. This way, the measured phase response from the PSDs is only affected by the SUT.

#### Square to sine spectroscopy algorithm

Now, as can be seen from Eqs. (5) and (6), it is not trivial to recover the impedance magnitude and phase of the fundamental when using PSDs, as is the case with LIAs. Indeed, harmonics of the square excitation signal are present at every odd frequency of the fundamental which adds a systematic error to the impedance measurement^54,56^. The harmonics present in the square signal are multiplied together by the mixer and pushed to DC along with the desired fundamental frequency. This systematic error is non-trivial as it depends on the impedance response of the SUT.

An algorithm inspired from Subhan^54^ can be used to cancel the systematic error. The values of square impedance at the harmonics can be subtracted or added to the fundamental impedance following a certain set of rules described in Subhan^54^. It is thus necessary to measure the entire impedance spectrum before computing the corrected impedance at a given frequency. The real component of the impedance devoid of the systematic error $V_{sine-\omega _0}$, follows Eq. (7)

7 $$\begin{aligned} \Re (V_{sine-\omega _0}) = \displaystyle \sum _{n=1,3,5,7...} ^{\infty } \frac{\cos (\theta (n \omega _0))}{n^2|Z |(n \omega _0)} - \displaystyle \sum _{n=3,5,7...} ^{\infty } \frac{\cos (\theta (n \omega _0))}{n^2|Z |(n \omega _0)} = \frac{\cos (\theta (\omega _0))}{|Z |(\omega _0)} + E \end{aligned}$$

where *E* is the residual error after correction, which depends on the number of frequency points that were subtracted. There is, however, a practical limit to the number of points that can be subtracted considering that the impedance at that frequency must be measured (or extrapolated) beforehand, which might not be possible for high frequency samples. A similar process can be repeated for the quadrature component in Eq. (8). The corrected impedance magnitude and phase of Eqs. (9) and (10) can then be reconstructed.

8 $$\begin{aligned} \Im (V_{sine-\omega _0})= & {} \frac{\sin (\theta (\omega _0))}{|Z |(\omega _0)} + E \end{aligned}$$

9 $$\begin{aligned} |Z(\omega _0) |= & {} \frac{1}{2} \frac{6}{\pi ^2} \frac{ V_0 R_f}{\sqrt{(\Re (V_{sine-\omega _0}))^2+(\Im (V_{sine-\omega _0}))^2)}} \end{aligned}$$

10 $$\begin{aligned} \theta (\omega _0)= & {} -\arctan (\frac{\Im (V_{sine-\omega _0})}{\Re (V_{sine-\omega _0})}) \end{aligned}$$

#### Printed circuit board

Considering that the microparticles that pass in the channel are microscopic, the sensor has to possess a high sensitivity. For the electronics, a thorough understanding of noise and best PCB design practices is required. The impedance-sensing system is made from a four-layer PCB, and has a size of 50 $\times$ 50 $\times$ 15 mm including the components. The substrate is FR-4 TG150, with minimum spacing of 0.1524 mm and a thickness of 1.5 mm. Finally, the surface finish is HASL with 1 oz copper. The final PCB with all components is shown in Fig. 2.

#### Microcontroller unit

The IFC system uses a MSP430F5529 as microcontroller unit (MCU). The MSP430F5529 is a mixed signal MCU used in low-power applications. It dissipates about 6 mW when active and 24 µW when in low-power mode. 6 channels of the 12-bit ADCs are used by the impedance-sensing system to measure the real and imaginary values of the outputs of the phase sensitive detectors $\Re (V_{PSD-\omega _0})$ and $\Im (V_{PSD-\omega _0})$ of the two electrode responses, as well as the 5V power-supply voltage $V_{DD}$ and the battery voltage. The I2C module is used to program the clock-system and modify the excitation signal frequency. The UART module is used with the external integrated chip FT232RL to transfer the data to a nearby computer using Bluetooth or USB 2.0. A couple of I/O pins are used to enable the power-supplies and status LEDs, modify the gains of the PGA on the fly, reset the phase of the measurement by enabling or disabling the flip-flops of the quadrature generator, and reset the MCU.

### Microfluidics system

The microfluidics system created for this study encompasses the micro-electrodes designed on PCB and a PDMS microchannel squeezed hermetically between 3d-printed components.

#### Microchannel

**Figure 4:**
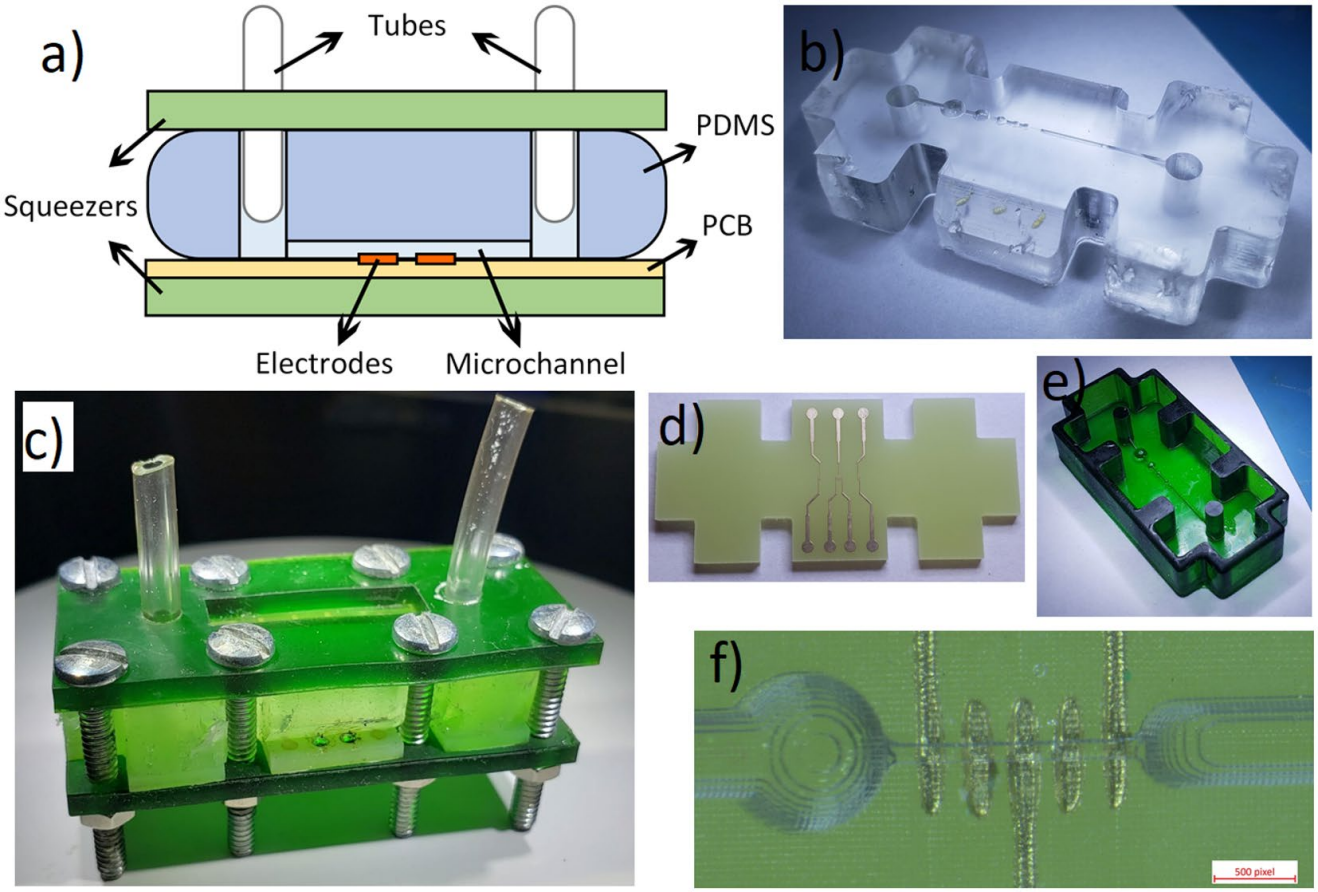
The different elements that compose the microfluidics system. (**a**) The diagram of the microfluidics system. (**b**) The PDMS microchannel. (**c**) The entire microfluidics system. (**d**) The PCB electrodes. (**e**) The 3D-printed mold used to cast the microchannel. (**f**) The aligned PDMS microchannel on the PCB electrodes.

The microfluidic system manufactured in this study has an inlet and an outlet, where the liquid respectively enters and exits the device, fitted to soft Tygon thermoplastic tubing^57^. The inlet tube is linked to a glass syringe connected to a precise motorized syringe pump by Cole-Parmer model CP-120 that compresses the syringe at a constant programmable rate. The SUT flows from the syringe to the tubes before entering the inlet. It then reaches the PDMS microchannel where it is sensed by the PCB microelectrodes. The liquid finally exits through the outlet tube, which is connected to a waste container. The microfluidics system is shown in Fig. 4.

The whole fabrication process is described in Bouzid^38^. A mold is initially drawn on a CAD software such as Solidworks. The drawn model is sent as a .STL file to the CADworks3D software to be meshed. This new meshed model is used by the stereolithography 3d-printer CADworks3d H50-405 to print a 3D-mold using Master Mold for PDMS Device Photopolymer Resin ^TM^. The resin is then rinsed with IPA (90%) or methyl hydrate for 5 min and blow dried using an air gun. The mold is then cured using UV light around 400 nm in a LED light curing box for 50 min. PDMS (SYLGARD^TM^ 184 Silicone Elastomer Base) and a curing agent (SYLGARD^TM^ 184 Silicone Elastomer Curing Agent) are mixed at a ratio of 10:1 and degassed by letting the solution rest for 60 min. The mixture is put in the mold and cooked on a hot plate for 50 min at 70$^{\circ }$, then dried overnight at 40 $^{\circ }$C^58^. A scalpel is used to gently prick-off the channel from the mold. The surfaces of the PDMS microchannel are then exposed to plasma at 600 KPa for one min. Polyethylene glycol is immediately applied on the surface to keep its hydrophilicity for longer periods of times. After a 10 min wait time, the PDMS microchannel is cooked for 10 min at 130 $^{\circ }$C on a hot plate^58^. A dry gun is blown on the channel to take away any residues. The PCB electrodes are aligned on the microchannel and a pressure is applied to seal them. The PDMS channel and PCB electrodes are sealed tight by a system of 3d-printed squeezers that compress the channel and PCB electrodes. Those are tightened together by bolts, hermetically sealing the microchannel and PCB electrode due to the flexible nature of PDMS. Finally, tubes are inserted at the inlet and outlet of the PDMS microchannel, concluding on the whole process. The microfluidics system is thus hermetic and easy to handle.

The shape of the mold is shown in Fig. 4. The volume fraction (i.e. the ratio between the volume of the microparticle and volume of the fluid affected by the electrical field of the electrodes) should be maximized in IFC applications to obtain the highest sensitivity. Maximizing the volume fraction requires that the channel and electrodes size be identical to the microparticle of interest. In practice, the channel must be larger by a given safety factor to allow the fluid to circulate without clogging. Hemispherical bubble traps based on the work of Kang^59^ were added to the channel to reduce the quantity of air bubbles reaching the electrodes which could falsify the measured impedance. Using the stereolithography 3d-printer H50-405, a theoretical resolution of 30 µm is possible, although in practice, the minimum size of a printable channel without major defects is 90 µm. For the case of this study, a channel size of 180 µm was chosen. The PDMS cured in this mold solidifies into a structure with four openings where are placed the bolts. The electrode and squeezers share the same shape and openings for the bolts as the PDMS.

#### Electrodes

Coplanar electrodes are chosen in this design because they are 2D structures that can easily be made using a lithography mask or directly on a PCB^28^. The non-homogeneous electrical field distribution of coplanar electrodes does introduce errors in the measurement since the cell’s vertical position in the channel are subject to varying levels of electrical field. A higher particle in the channel typically experiences weaker electrical field than a low particle, which results in a lower perceived amplitude^51,52^, as was shown in Fig. 1. Since the amplitude of the spike is used to infer the cell properties, a significant error is thus observed. Three solutions can be used to counter this problem: (1) Using parallel facing electrodes placed diagonally opposed in the channel instead of coplanar ones^52^. (2) Using centering techniques such as dielectrophoresis, acoustophoresis, inertial focusing and sheath flows^51^. (3) Using coplanar electrodes with distinctive geometry to obtain additional information about the vertical position of the particle in the channel^41,51^. The 5-electrode configuration described in De Ninno^51^ is one of those distinctive geometry and has been chosen for this study, and is shown in Fig. 2. The relative prominence of the signal obtained from such a configuration can be used to correct the measured particle size. The downside of such a technique is that it reduces the sensibility of the sensor since the intricate electrode geometry increases the sensing volume^60^.

The electrodes in this study are fabricated on a one-layer PCB. They have a size of 46 $\times$ 21 $\times$ 1.6 mm. The PCB uses the conventional substrate FR-4 TG130 in the exact shape of the microfluidics channel. The employed surface finish is immersion gold (ENIG) (1U”) with 1 oz copper. Inert metals such as gold or platinum are used for the electrodes because of their convenience in casting for small dimensions, for their unlimited lifetime, and since other types of electrodes such as Ag/AgCl are unsuitable for high excitation frequency^28^. The electrodes are 101.6 µm wide and are separated by 101.6 µm each. The PCB electrodes and their alignment with the microchannel are shown in Fig. 4.

## Results and discussion

To test and calibrate the performances of the device, EIS analysis of discrete resistors and capacitors forming complex circuits were measured. EIS analysis on saline water, as well as the detection and characterization results of microbeads and buccal cells using IFC also follow. Finally, the performance of the system is discussed.

### Complex impedance circuit

**Figure 5:**
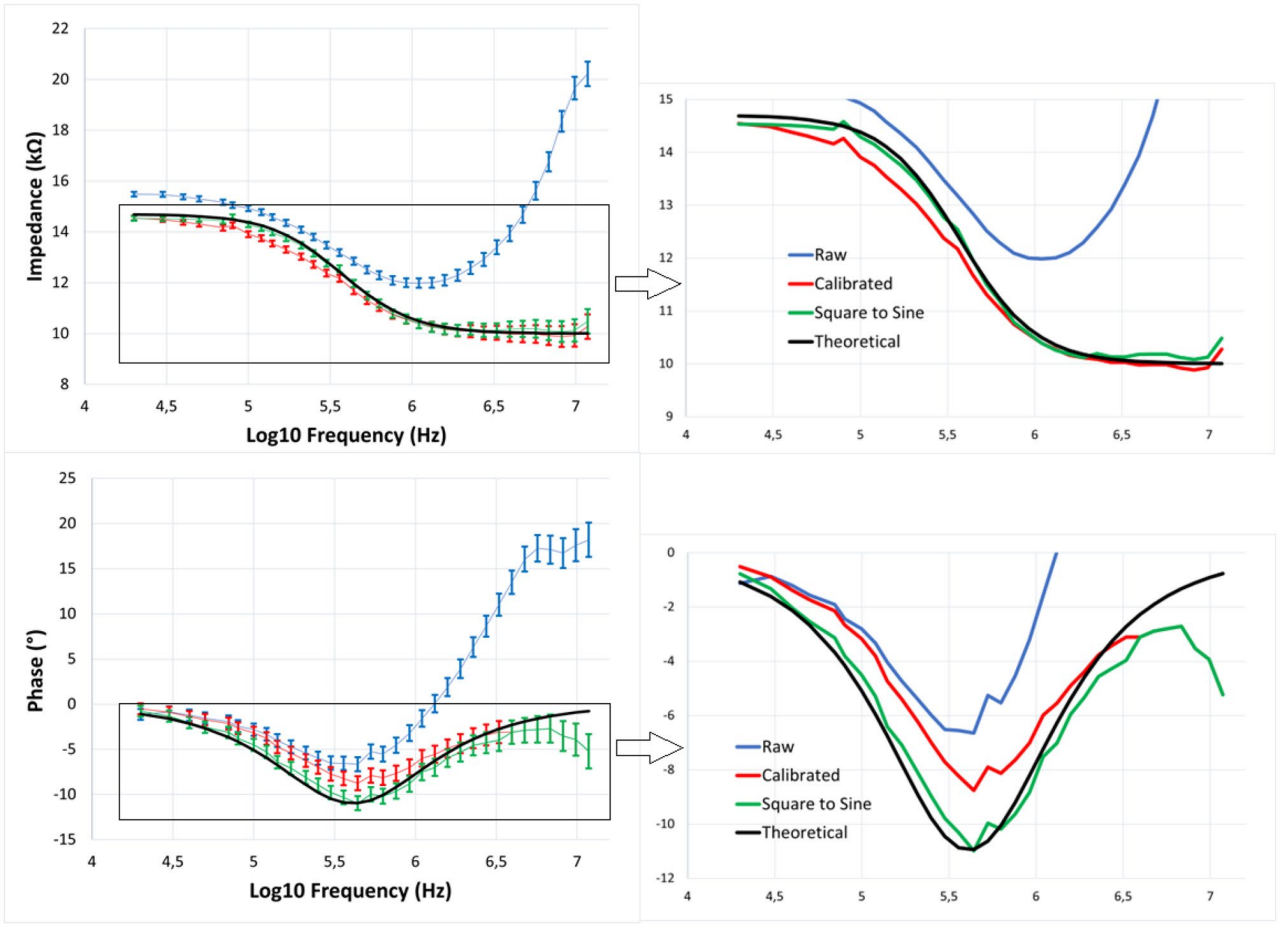
Bode plot of the impedance magnitude and phase response of a 10-k$\Omega$ discrete resistor in series with the parallel combination of a 4.47-k$\Omega$ resistor and a 100-pF capacitor. 320 samples were taken from the SUT for each frequency, and the average and standard deviations are calculated and displayed on the error bar on the left. Four sets of data are displayed, the measured raw impedance, the raw impedance after calibration, the calibrated impedance after transformation using the square to sine spectroscopy algorithm, and the theoretical impedance of the SUT.

The performances of the impedance-sensing system are measured from the EIS analysis of a 10 k$\Omega$ discrete resistor in series with a parallel combination of a 4.7 k$\Omega$ resistor and a 100 pF capacitor. The impedance-sensing system has a lowest excitation frequency of 20 kHz and a highest of 12 MHz. The square excitation signal is initialized at the lowest frequency, samples 64 data points, then the frequency is incremented logarithmically until the end frequency is reached. When that is the case, the frequency is reinitialized to the lowest frequency, and the process begins anew. The impedance magnitude and phase are shown in Fig. 5, and were recorded for about 34 s at a sampling rate of 655 Sps, which amounts to about 320 data points per frequency.

A lower accuracy and precision can be observed for low and high frequency. This is to be expected since the frequency range of the impedance-sensing system is defined only for frequencies above 100 kHz due to the LPF of the PSDs having a $-3$ dB low-pass cutoff frequency of 20 kHz, which does not totally attenuate the square excitation signal up until at least 70 kHz. The high frequency bias originates from multiple sources. Firstly, the op-amps used in the electronics of the numerous modules are limited in frequency by their slew-rates, input capacitance, and gain-bandwidth. Secondly, capacitive coupling is introduced because of the PCB dielectric, traces, and wires, which tend to attenuate the high-frequency components. Since square excitation signals are used, the harmonics at higher frequencies get attenuated until the signal behaves much more like a sinewave around 20 MHz. As a result, the perceived impedance from the impedance-sensing device is increased since the measured current response is attenuated at high frequency.

To solve some of these issues and linearize the sensor, a calibration using a look-up table is realized using resistors of known values. Since the parasitics are singular to the electronics, the same nonlinearity will be found for different values of resistance, which can be used as a frequency-dependent factor to linearize the magnitude and phase curves. The square-to-sine conversion adapted from Subhan^54^ is then performed on the calibrated dataset. The raw, calibrated and converted-to-sine results are shown in Fig. 5 for the median impedance at each frequency points. At high frequencies, a bias is observed both in the impedance and phase since higher frequency data points are not available to perform the square to sine conversion from Eqs. (7) and (8). A way to solve this issue would be to extrapolate the behavior of the system from the previous points and use that extrapolation in the square to sine conversion. For the sake of simplicity, no such correction was attempted in this study. Apart from that bias, errors of less than 3% are observed for the magnitude and phase, for the frequency range considered in the spectroscopy. This is comparable to the commercial devices presented in Table 1, at a fraction of the power consumption and cost.

### Saline solution

Following the proof of functioning and the calibration, we measure the impedance spectrum of a complex system. A solution of saline water at 22 $^{\circ }$C is passed in the 180 µm wide PDMS microchannel of the microfluidic system, and the EIS is measured. The calibrated and converted EIS curves for the two pairs of electrodes follow the behavior of a series capacitor and resistor, as expected from the Randles model. This dataset will be used to calculate the corrected impedance after using the square-to-sine transformation algorithm after the addition of microbeads and buccal cells.

The measured impedance varies slightly according to the pressure exerted by the liquid flow. This difference is caused by a slight contraction of the PDMS walls caused by the liquid pressure, which also increases the liquid volume measured by the electrodes. The same effect can be observed for variations in temperature of the liquid. Thus, the liquid pressure and temperature are controlled for the whole duration of the experimentation.

### Microbeads

In order to replicate more accurately the expected behavior from cells and microparticles, polyethylene microbeads are added to the previous saline solution. This SUT is kept at the same conditions as before, at 22 $^{\circ }$C and passed in a 180 µm wide microchannel. Only one square excitation signal is used this time, at a frequency of 1 MHz. The corrected impedance can then be calculated with Eq. (7) using the measured impedance of the microbeads as fundamental and the EIS of saline water as the harmonics. Considering the small difference in impedance spectroscopy between both tests, errors less than 1% are expected.

To avoid overloading the MCU, the impedance is sampled at a fixed high-frequency rate of 5461 Sps, while data is saved to memory only when a significant difference is observed in the real parts of the measured impedance of either electrodes. When that condition is detected, a burst of 64 consecutive measurement points is saved. This method produces regularly fixed data point with dense bursts of data when an event is detected. This event detection can be caused by a microparticle passing in the channel, or by sudden changes in liquid property or microchannel geometry. Signal processing is performed offline to retrieve only the events associated with a particle detection. Firstly, the average of the magnitude difference is removed using wavelet decomposition, the signal is then de-noised, low-pass filtered, and smoothed so that the impedance spikes caused by the particles are easier to recognize. A particle detection algorithm is used on this dataset to recover the positions of the peaks. A peak detection algorithm is first used, followed by a weak supervision approach using Snorkel^61^ to discriminate between the peaks obtained from microbeads, bubbles, or any other outliers. Most of the oddly shaped, or weird behaving particles are thus removed from the dataset automatically. With the particles peak locations, it is possible to recover the amplitude and width of the patterns, which are used to estimate the microbeads size using Eq. (2).

**Figure 6:**
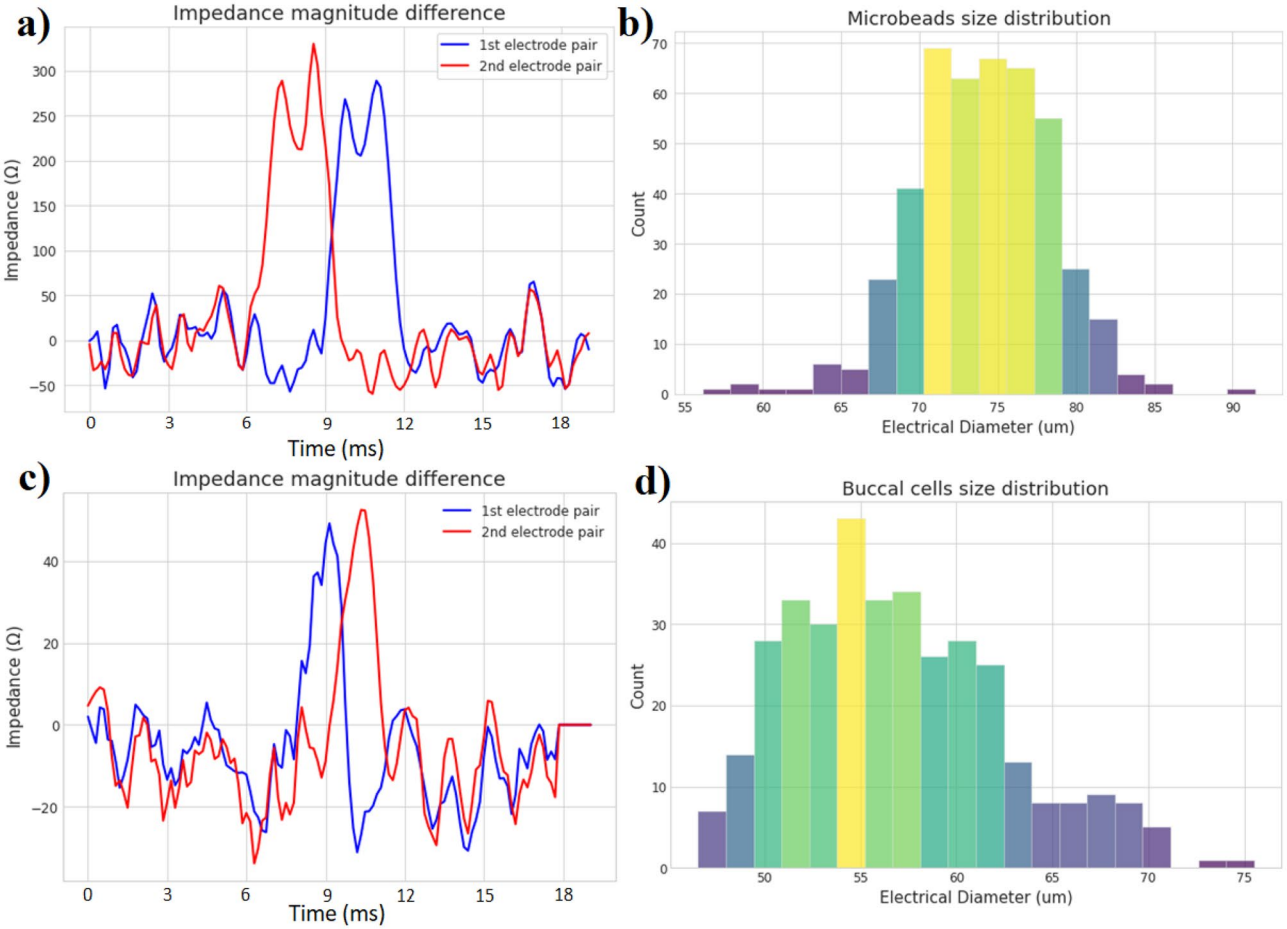
Magnitude difference of both electrode pairs (**a**) when a 78 µm polyethylene microbead passes in the 180 µm wide microchannel. (**c**) when a 51 µm buccal cell passes in the 180 µm wide microchannel. Distribution of (**b**) the 63–83 µm microbeads population and (**d**) the buccal cells population.

As an example, the pattern in (a) of Fig. 6 is studied. The first electrode has an impedance magnitude difference of 230 $\Omega$, while the second electrode has an impedance magnitude difference of 250 $\Omega$. Its *G* constant is estimated from the dataset to be around 10. This leads us to a diameter value of 78 µm. It is also possible to measure the particle velocity by dividing the distance between the electrode pairs *L* with the time it took for the particle to go from one electrode to the other $\Delta t$ (which is the time difference between the two impedance maximums). The time it took for the particle to pass the electrodes is found to be 2.9 ms, while the distance between the electrode pairs is of 406 µm. This leads to a velocity around 14 cm/s. This flow rate has been found empirically to provide good and reliable measurements, since lower flow-rate can cause particles to stick to the walls of the channels or electrodes, and higher flow-rate are associated with a decreased time resolution. The fastest useful flow-rate for this sampling rate is when a minimum of 5 points are detected for a full particle. Any less than that is considered an outlier by the classifier. This leads to a maximum theoretical flow-rate of 89 cm/s.

### Buccal cells

The proposed system works to detect cells of a maximum size fixed by the width of the microchannel, and of a minimum size fixed by the sensibility and inherent noise of the sensor. For the case of this study, this leads to a minimum and maximum cell size of 45 $\upmu m$ and 180 $\upmu m$ respectively. An easy to test cell that fits those size requirements are those found in the mouths, the so-called buccal cells, with sizes typically ranging between 50 and 60 $\upmu m$^62^. Those cells were scraped from the tongue and cheek of the corresponding author, and mixed with the same saline solution used in the previous tests. An example of a cell detection is shown in Fig. 6, where the 1st and 2nd pair of electrodes each detected successive impedance events, which were used to characterize the cell size around 51 µm.

### Collected datasets

The true potential of IFC sensors lies in how automatizable the sampling and testing process can be for biological studies. It could be imagined that a team of biologists collects the impedance data of millions of cells and particles using the portable device described in this study, and efficiently extract the important information of these cells using machine learning and high-end signal processing. As a small-scale example, two datasets were collected from the proposed system and passed into the peak detection and classification algorithm. 447 beads were detected from 617 detection events, and 360 buccal cells were successfully detected from 2823 detection events. The debris in the saline solutions from the cheek and tongue scraping, and the low impedance difference obtained from the buccal cells explain why so much detection events were detected by the algorithm compared to the number of actual buccal cells. The diameters of the microbeads and buccal cells were estimated as described previously and compiled in the histograms of Fig. 6. A minimum impedance difference threshold is used to classify what counts as a detection event from the measurement noise. This lower threshold means that the microparticles of sizes below 45 µm that are present in the solution are not registered by the algorithms. It could be said that the effective sensitivity of the impedance-sensing system to detect small particles is of 45 µm when used with a 180 µm wide microchannel.

### Measured performance

**Table 2:** Measured system performances.

Parameter	Value
Supply voltage (battery)	2.5–3 V
Power consumption	1.2 W
Input voltage @ electrodes	100 mV
Sampling rate	5461 Sps
Excitation frequency	30 kHz–12 MHz
Type of excitation signal	Square
Impedance magnitude precision	< 3%
Impedance phase precision	< 3%
PCB size	50 $\times$ 50 $\times$ 15 mm
Microfluidics system size	46 $\times$ 25 $\times$ 50 mm
Total device weight	300 g
Microchannel area	180 $\times$ 180 µm
Electrode width	106 µm
Electrode pairs separation	424 µm

The performance and characteristics of the presented impedance-sensing device and microfluidic system are summarized in Table 2. The impedance-sensing system created for this study is the first found in the scientific literature to achieve great sensitivity level over wide frequency and impedance range while boasting a small size, low-cost, and low power-consumption. The impedance-sensing device coupled with the microfluidics systems are effectively capable of measuring and estimating the properties of the microparticles of sizes going as low as 45 µm when used within a 180 µm wide microchannel. The dimensions of the microchannel are fixed by the limitations of the 3D-printer, which could be improved for this study by using a 3D-printer such as the one designed by Gong^63^. This homemade 3D-printer is specifically made for microfluidics and can attain truly microscopic scales of 18 $$\times$$ 20 µm by modifying the type of resin used and optimizing the stereolithographic process. This higher resolution would help increase the sensitivity of our device for smaller particle detection. The impedance-sensing device takes 50 mm $$\times$$ 50 mm $$\times$$ 15 mm of space, while the microfluidics system is 46 mm $$\times$$ 25 mm $$\times$$ 50 mm, with a combined weight of 300 g, making them portable enough to be put in a backpack for applications in the field. The electrode pairs in the microchannel are separated by 424 µm and each have a width of 106 µm compared to the microchannel size of 180 µm $$\times$$ 180 µm. The impedance-sensing system only needs 1.2 W to function adequately, and is powered by a low-voltage battery of 2.5–3 V. The power consumption of the system is sufficient for portable applications and could be powered for a couple of hours at a time. The power consumption could, however, be greatly reduced by creating a custom ASIC instead of using discrete components. Indeed, the vast majority of the power (about 80%) is dissipated in the op-amps, while they serve only to do basic functions such as inverting and amplifying signals that could be replaced by optimized high frequency transistors. The impedance-sensing system costs around $300, while the microfluidics system costs only $10 per microchannel excluding the initial cost of the 3D printer. The impedance measurement range between 200 $$\Omega$$ and 120 k$$\Omega$$ is similar to the portable impedance analyzer described in the literature, such as the ones from Al-Ali^37^, and Radil^34^. The frequency range is adequate for IFC applications, with the important frequency range between 100 kHz and 10 MHz covered by the device. The upper frequency limit of 12 MHz observed in this work is fixed by the limitations of the op-amps used in the TIAs. The limited bandwidth of the op-amps attenuates the harmonics of the square signal, which progressively modifies the square excitation signal into a sinusoidal shape. This introduces significant disparity for frequencies higher than 12 MHz which goes above the 3% precision reported for the device. The device can theoretically be used with excitation frequencies as high as 100 MHz, but the reported error would increase significantly. Finally, an excitation voltage of only 100 mVpp is used, which is low enough to not affect most microorganisms in that size range.

## Conclusion

This study succeeded in creating an autonomous device for the characterization of microorganisms in the fields. Using an inexpensive 3D printing manufacturing technique and standard printed circuit board technology, the presented device can detect and characterize microorganisms larger than 45 µm. The device succeeded in characterizing and differentiating between buccal cells and polyethylene microbeads. Future work will focus on improving the sensibility of the sensor to characterize microparticles of smaller sizes, as well as increasing the number of parameters that can be monitored to achieve a better characterization. Following the recent advances in micro-optical systems, adding a low-power 3D-imaging system to the device will be investigated.

## Data Availability

The Python, MATLAB, and C source code for this project, and the dataset obtained from the device are available upon reasonable request to the corresponding author. Python was used to sample the dataset from the device. C code was used to interact with the sensors and MCU. Post processing was done in Matlab and Python.
